# Social Determinants of Health and Chronic Kidney Disease

**DOI:** 10.7759/cureus.10266

**Published:** 2020-09-05

**Authors:** Jessica Quiñones, Zeidan Hammad

**Affiliations:** 1 Biological Sciences, Herbert Wertheim College of Medicine, Florida International University, Miami, USA; 2 Humanities, Health and Society, Herbert Wertheim College of Medicine, Florida International University, Miami, USA

**Keywords:** ckd disparities, social determinants of health, chronic kidney disease, primary care physician

## Abstract

In the United States, chronic kidney disease (CKD) remains a concern to the health of 37 million adults. Prevention is key to combatting this disease before it is able to progress into its advanced stages or end-stage renal disease (ESRD). Awareness of the clinical early detection of CKD is important for healthcare providers, but a look at the causes of this disease can provide for greater evaluation of the patient’s risks. Understanding of social determinants of health (SDOH), the non-clinical aspects of a patient’s life that affect disease onset and progression, and its effects on CKD and ESRD should be greater emphasized in the primary care field. Population groups living with relatively poorer SDOH are disproportionally affected by CKD and ESRD. Although political change is out of the scope of the primary care health provider, efforts in combatting poor social determinants can provide for better outcomes for at-risk or diagnosed patients. Education and screening are two suggestions brought up in literature for tackling SDOH, specifically in the primary care settings most applicable to CKD.

## Introduction and background

As of 2019, the Centers for Disease Control (CDC) approximates that 15% of adults in the United States have chronic kidney disease (CKD) [1]. In the 37 million people estimated to have CKD, disparities related to socioeconomic status, gender, race, and social environment have been increasingly highlighted as key elements in CKD risk, progression, and onset of complications. The growing global recognition of social determinants of health (SDOH), defined as the “conditions in which people are born, grow, live, work, and age,” is key for better understanding of outcomes and public health. With the United States falling behind other developed nations in preventable deaths, coupled with growing poverty and other adverse SDOH, action must be taken to mitigate health disparities [2]. Primary care physicians are central to identifying clinical factors to disease, but with evidence that SDOH influence health outcomes more than medical care, these nonclinical factors should be greater emphasized [3].

## Review

SDOH relating to CKD disparities

CKD is more commonly seen in non-Hispanic Black (16%) and Hispanic (14%) people, compared with non-Hispanic White (13%) and non-Hispanic Asian (12%) people [4]. Although historically, these racial disparities have been attributed to genetics, the imprecise nature of socially defined racial and ethnic groups should be seen as a nonclinical determinant. Black and Hispanic individuals are, on average, in a lower socioeconomic status than White individuals [5]. When it comes to socioeconomic status, several studies have correlated lower socioeconomic status to higher prevalence and incidence of CKD in communities, even when controlling for other social factors [6]. Along with lower income, Americans with fewer than 12 years of education were also found to have greater prevalence of lower kidney function [5]. The two major causes of CKD, diabetes and hypertension, are also subject to similar disparities due to social determinants [3]. Diabetes, afflicting 285 million adults worldwide, affects Black and Hispanic people at greater percentages than White people in the United States [7].

The same disadvantageous circumstances that may have influenced the onset of CKD in individuals is usually still present after diagnosis. These social determinants worsen the quality of life and progression of the disease. For example, one of the recommended treatments a primary care physician might recommend, in agreeance with CDC guidelines, would be an improvement of lifestyle and diet. However, adults with CKD experience a higher rate of food insecurity than the general population at a rate of one in four [8]. Food insecurity is associated with unhealthier diets, which is then connected to higher rates of diabetes, hypertension, and CKD. These increasing adverse conditions such as food insecurity, low income, and lack of education have been found to correlate with increased mortality [9]. Globally, socioeconomically disadvantaged populations that experience the heavier burden of CKD deal with the negative complications that similarly prohibit them from receiving proper, evidence-based care that could improve their clinical outcomes [10].

Eliminating the SDOH mentioned such as poverty, race, and food insecurity reach beyond the scope of a health care provider or the field of medicine. To eradicate these factors, changes in policy and infrastructure are in order. The World Health Organization has identified key tenets to improving global health through ameliorating SDOH, such as tackling inequal distributions of power and resources [11-12]. Policies relating to socioeconomic equality aim to reduce the systemic barriers that maintain the disadvantageous circumstances that affect health outcomes. Although these broader strides towards change may seem abstract and outside of the scope of medical practice, that does not mean that the SDOH should not be acknowledged in the clinic. With the growing disparities in health, efforts to combat the SDOH are also expanding. The idea of merging public health with primary care is not new, but increasing disparities signal for a need for greater emphasis than current measures [13]. This includes accessible policies and movements to improve CKD prevention and care [14-17]. Although it is generally agreed that these determinants exist, what to do from this point is often contended. In order to address the SDOH from a primary care point of view, a look at the sprouting ideas will be explored in this review. including introduction during medical education and possible screening for SDOH in practice.

Addressing SDOH through primary care

For further integrating SDOH in practice, future physicians and healthcare workers should be appropriately educated about social and economic disparities in health by the school of medicine. Currently, there is a gap in the literature regarding SDOH being incorporated into the graduate-level medical curriculum [18]. The scarcity of standardized tools and strategies for institutions to use inevitably results in an unequal knowledge base on SDOH for health care professionals [18]. For example, some programs offering SDOH classes as optional to their students show the inconsistencies of the subject in curricula [19]. Making the course work universal and moving SDOH from elective to mandatory would increase the emphasis of its relevance in practice [19-22]. Incorporation of the curricula into clinical education will also allow students to see the relevant applications in practice, such as interdisciplinary communication [19-21]. While it may be unrealistic to assume that proper education would solve SDOH disparities, promoting the learning of the material may aid future healthcare workers in understanding the social context of their patient’s health to address those challenges properly. In a study with post-graduates year-1 (PGYs-1), brief group sessions for introducing SDOH concepts to internal medicine residents found that residents that were taught these concepts were more likely to believe that physicians have a responsibility to address SDOH in clinical settings, they just did not know how. In this case, faculty development was key to prompting trainees to identify SDOH in patients, understanding the best interventions, and using community resources available to intervene [22].

Another suggestion for handling SDOH is screening for them in the clinic [23]. Currently, the few social screenings recommended by the U.S. Preventative Services Task Force are for singular issues, such as screening for domestic violence in young women, which have been largely effective [24]. Although there isn’t much literature on screening for general adverse SDOH in primary care, there are some budding programs exploring this idea. Examples of initiatives that aim to alleviate inequalities in vulnerable communities are the National Kidney Foundation Early Evaluation Program, mobile clinics, increasing health literacy through CKD videos and/or pamphlets, and intervention from patient navigators [25-28]. Similar models such as WE CARE were able to successfully implement electronic health record automation to facilitate screening and referrals, resulting in 86% of patients who requested assistance to receive a relevant guide to getting adequate resources, such as, screening done by medical assistants to relieve physician burden. To increase efficiency, automated machine learning tools have been utilized to identify at-risk patients of adverse SDOH using available data, improving patient attendance for future clinic appointments. These results showed that implementing screening tools with electronic health records is feasible in an urban safety net primary acre setting [29]. In this setting, link workers can be used to refer patients to internal health system resources or other outside social services. Resources such as the CLEAR (Community Leadership on the Environment, Advocacy, and Resilience) toolkit, an electronic SDOH aid, or comprehensive lists such as the multideterminant Health-Related Social Needs Screening Tool from the centers for Medicare & Medicaid Services use can be utilized. When social needs are found, there are other tools to connect like the American Academy of Family Physicians Neighborhood Navigator that can find local resources to help. If a practice establishes a partnership with community organizations, care for recurring social needs can be better facilitated [30]. It is also important to note that most research about these patient-level strategies focuses more on immediate process outcomes and not impact on health outcomes, requiring the need for expansion on optimal strategies and their effects [24,31]. This concept still requires exploration, and some clinicians feel that screening for SDOH will take time away from their patients and that they are not equipped to effectively handle the issues. Additionally, another screening tool would place a higher burden on the healthcare worker to complete another administrative duty. Ideally, an automated system would be able to flag or refer a patient without too much work from the clinician [22,29].

It is the current view of the authors that although the literature on the effectiveness of SDOH intervention still requires expansion, these changes can prove to be useful with patient care and can aid in improving patient outcomes. Although current measures do not seem to be adequate yet, an emphasis on social determinants has been gaining traction with the initiatives described above. This does not imply that it is the physician’s job to enact policies that enforce socioeconomic-aware practices, but to be informed about SDOH and its effects in practice [22]. Before recommending routine screening for social needs, an accurate screening test to identify the patient, an effective treatment to address the social need, and evidence that this improves patient outcomes is needed. Literature accurately proves that social needs lead to worse outcomes, but what healthcare professionals do to address the issue is vaguely recorded. Despite the lack of experimental data on screening effectiveness or SDOH education on patient outcomes, there is a growing enthusiasm with these types of proposed solutions, and greater implementation by healthcare systems to attempt to address SDOH are being put in place [23].

## Conclusions

Adverse social determinants of health have been shown to have a profound effect on the health of the individuals, including for CKD and subsequently, end-stage renal disease (ESRD). It is crucial that all stakeholders unite in the initiative to manage both the clinical and non-clinical aspects of CKD to effectively provide the best care of afflicted patients. This includes recognizing the social factors that contribute to disease, as this is increasingly important for patients that are in vulnerable communities whose social needs have largely been ignored. 
